# Dual-Algorithm Integration Framework Reveals Qing-Wei-Zhi-Tong’s Dual Mechanisms in Chronic Gastritis

**DOI:** 10.3390/ph18111743

**Published:** 2025-11-17

**Authors:** Zhijie Shu, Ying Huang, Yujie Xi, Bo Zhang, Rui Cai, He Xu, Feifei Guo

**Affiliations:** 1 State Key Laboratory for Quality Ensurance and Sustainable Use of Dao-di Herbs, Institute of Chinese Materia Medica, China Academy of Chinese Medical Sciences, Beijing 100700, China; shuzj0619@163.com (Z.S.);; 2 Experimental Research Center, China Academy of Chinese Medical Sciences, Beijing 100700, China; 3 Jiangxi Province Key Laboratory of Traditional Chinese Medicine Pharmacology, Institute of Traditional Chinese Medicine Health Industry, China Academy of Chinese Medical Sciences, Nanchang 330115, China

**Keywords:** Qing-Wei-Zhi-Tong micro-pills, gastritis, dual-algorithm integration framework, similarity based heterogenous network, network disturbance

## Abstract

**Background**: Chronic gastritis (CG) involves gastric mucosal imbalance, with *H. pylori* (>90% cases), acid-pepsin imbalance, and bile reflux as druggable mechanisms. FDA-approved drugs show limited efficacy against antibiotic-resistant strains and fail to target undruggable pathways (e.g., inflammation, autoimmune atrophy). Traditional Chinese Medicine (TCM), particularly Qing-Wei-Zhi-Tong micro-pills (QWZT), offers multi-target advantages, though its mechanisms remain poorly understood. **Methods**: The dual-algorithm integration framework predicts QWZT’s pharmacological effects to treat gastritis. For druggable processes (pathways targeted by existing drugs), the structure–target–pathway similarity algorithm quantifies QWZT similar activities to FDA drugs, validated by gastrointestinal smooth muscle experiments. For undruggable processes (novel biological mechanisms not addressed by current therapies), the multi-target perturbation algorithm predicts QWZT’s unique capacity to undruggable processes and is validated via LPS-induced inflammation in RAW264.7 and GES-1 cells. **Results**: Structure–target–pathway similarity algorithm identified QWZT compounds sharing prokinetic mechanisms with FDA drugs, validated by dopamine-induced relaxations and acetylcholine-induced contractions in gastrointestinal smooth muscle. Multi-target perturbation algorithm quantified QWZT’s superior disruption of undruggable immune/inflammation networks, confirmed by restored cell viability in LPS-injured GES-1 cells and significantly reduced the expression of NO, IL-6, and TNF-α in RAW264.7 cells via key compounds (paeoniflorin and berberine). **Conclusions**: QWZT may exert its regulatory effects on gastrointestinal smooth muscle by mediating muscarinic and dopamine receptor D2 (DRD2), and reduce the expression of NO, IL-6, and TNF-α to achieve anti-inflammatory effects, thereby effectively treating CG. The integration strategy that integrates algorithms and experiments to reveal the common and distinct mechanisms of QWZT compared to FDA-approved drugs, offering a novel approach for studying Traditional Chinese Medicine mechanisms.

## 1. Introduction

Chronic gastritis (CG) is a prevalent digestive system disorder resulting from an imbalance in the gastric mucosa [1]. It is considered one of the most common findings at endoscopy in the general population of China [2,3]. CG is a persistent inflammation of the stomach lining with complex and various factors. *Helicobacter pylori* infection is a major contributor to CG [4], damaging the gastric mucosa and triggering inflammation. The inflammation affects nerve supply and smooth muscle, causing abnormal contractions and reduced motility [5]. CG is a representative cause of gastric cancer [6]. As a result, early intervention is highly significant in decelerating the progression of gastritis and inhibiting epithelial cell lesions.

FDA-approved drugs recommended for the treatment of gastritis were guided by the “China Consensus on Chronic Gastritis” (2017, Shanghai, China) [2] and categorized, including proton pump inhibitors (PPIs, ATC code: A02BC), H2 receptor antagonists (H2RAs, ATC code: A02BA), prokinetic drugs (ATC code: A03FA), selective serotonin reuptake inhibitors (SSRIs, ATC code: N06AB), antacids and gastric mucosal protectors (ATC code: A02BX). However, FDA-approved drugs often cause adverse reactions and play a partial role in relieving symptoms [7,8]. And its superiority was not demonstrated in antibiotic-resistant patients [9].

QWZT is a prescription drug approved by the National Medical Products Administration (NMPA) of China, which was commonly used to treat gastric ulcer and chronic non-atrophic gastritis [10]. It is composed of five herbs, including *Coptis chinensis* Franch. (Huanglian), *Paeonia lactimicrobiota* Pall. (Baishao), *Bletilla striata* (Thunb.) Rchb. f. (Baiji), *Sanguisorba officinalis* L. (Diyu), and *Endothelium Corneum Gigeriae Galli* (Jineijin). QWZT’s therapeutic effect on gastric ulcers may be attributed to its ability to modulate the gut microbiota, alleviate inflammation by lowering mediator levels, and rectify metabolic imbalances [11]. Furthermore, it suppresses the expression of NLRP3 inflammasome-associated genes, potentially enhancing the treatment of chronic non-atrophic gastritis by regulating the innate immune response [12].

Mechanistic investigations of TCM formulas such as QWZT are limited by their compositional complexity, where interactions among multiple bioactive compounds obscure target identification and by the absence of standardized comparative frameworks. These frameworks are needed to systematically distinguish common and specific mechanisms between TCM and FDA-approved drugs.

In this study, we developed a dual-algorithm integration framework to predict the pharmacological effects of QWZT. By combining computational biology with experimental validation, we have effectively elucidated the mechanisms underlying TCM through two complementary approaches. (1) Druggable processes: the structure–target–pathway similarity algorithm quantifies similarity between QWZT compounds (e.g., palmatine, magnoflorine) and clinically approved drugs (e.g., prokinetics/SSRIs) to predict potential activity of QWZT compounds. (2) Undruggable processes: the multi-target perturbation algorithm constructs gastritis-associated disease networks (e.g., immune/inflammation subnetworks) and evaluates QWZT’s disruptive capacity on undruggable processes. This framework systematically delineates the mechanisms through which TCM intervenes in gastritis, differentiating druggable processes (by similarity analysis with approved drugs) from undruggable processes (via identification of unique network perturbations).

## 2. Results

### 2.1. Identification of Active Compounds in QWZT

**Figure 1:**
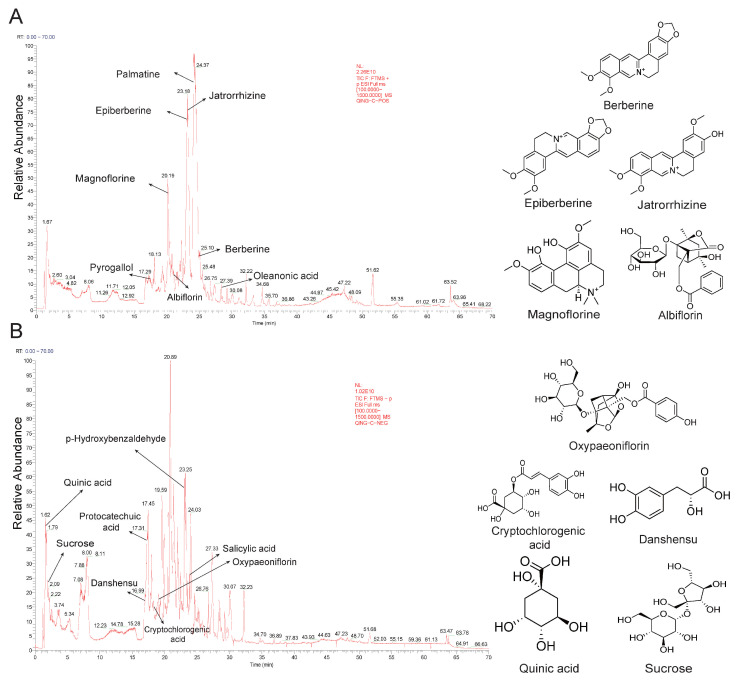
Identification of intestinal-absorbable compounds of QWZT. (**A**) Total ion current chromatogram (TIC) in positive ion mode, top 5 compounds in terms of peak area in this mode. (**B**) TIC in negative ion mode, top 5 compounds in terms of peak area in this mode.

Based on the UPLC–MS analysis technique and the everted intestinal sac model, 33 intestinal-absorbable compounds of QWZT were identified (Table 1), including Huanglian (14), Baishao (12), Diyu (5), Baiji (3), and Jineijin (1). In MS positive ion mode, 16 chemical compounds were identified, and the top 5 compounds with maximum peak area ratio were magnoflorine (23.06%), jatrorrhizine (16.93%), berberine (11.29%), epiberberine (9.40%), and albiflorin (2.64%) (Figure 1A). In MS negative ion mode, 17 chemical compounds were identified, and the top 5 compounds were sucrose (2.72%), quinic acid (2.66%), danshensu (2.27%), cryptochlorogenic acid (1.73%), and oxypaeoniflorin (1.41%) (Figure 1B).

### 2.2. Comparison of Potential Targets and Associated Biological Processes for QWZT Compounds and FDA-Approved Drugs

**Figure 2:**
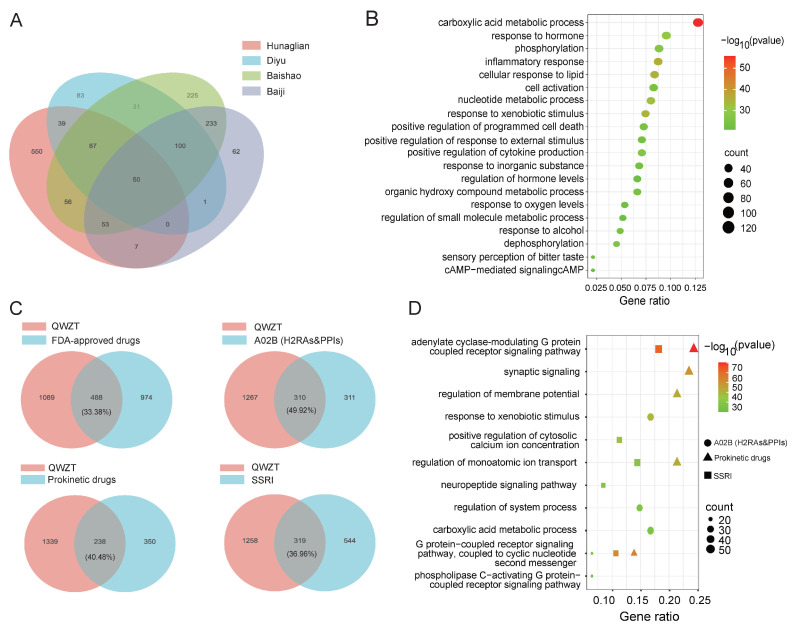
Comparison of target functions between QWZT and FDA-approved drugs. (**A**) Venn diagram shows the overlapping targets of 4 herbal ingredients of QWZT. (**B**) Top 20 enriched GO terms of QWZT targets. (**C**) Venn diagram shows the common targets of QWZT and FDA-approved drugs. (**D**) Top 5 enriched GO terms in the common targets of QWZT and FDA-approved drugs.

A total of 1577 potential targets for intestinal-absorbable compounds of QWZT were predicted from BATMAN-TCM, STITCH, and PubChem Bioassay database. Specifically, 842 targets of Huanglian, 835 of Baishao, 506 of Baiji, 391 of Diyu, and 1 of Jineijin (Figure 2A) were predicted. The GO enrichment analysis showed that the targets of QWZT were significantly enriched in carboxylic acid metabolic process, inflammatory response, and cellular response to lipid (Figure 2B).

The potential targets of FDA-approved drugs were predicted from LINCS, including 863 targets of SSRIs, 621 targets of A02B (PPIs and H2RAs), and 588 targets of prokinetic drugs. By comparing the overlapping of the targets in QWZT and FDA-approved drugs (Figure 2C), we found that A02B (49.92%) and prokinetic drugs (40.08%) exhibited a higher overlapping ratio with QWZT than SSRIs (36.96%). The common targets of QWZT and three types of FDA-approved drugs were mainly enriched in adenylate cyclase-modulating G protein-coupled receptor signaling pathway, positive regulation of cytosolic calcium ion concentration, and regulation of monoatomic ion transport (Figure 2D). At the same time, the targets of A02B were primarily focused on the metabolism of compounds, SSRIs, and prokinetic drugs enriched in monoatomic cation transport and regulation of membrane potential (Figure S3A–C).

### 2.3. Predicting Activity of QWZT Compounds Based on Structure, Target, and Pathway Similarity to FDA-Approved Drugs

To predict pharmacological activity of QWZT compounds via similar approved drugs, we proposed similarity evaluation to FDA-approved drugs using structure, target, and pathway data.

**Figure 3:**
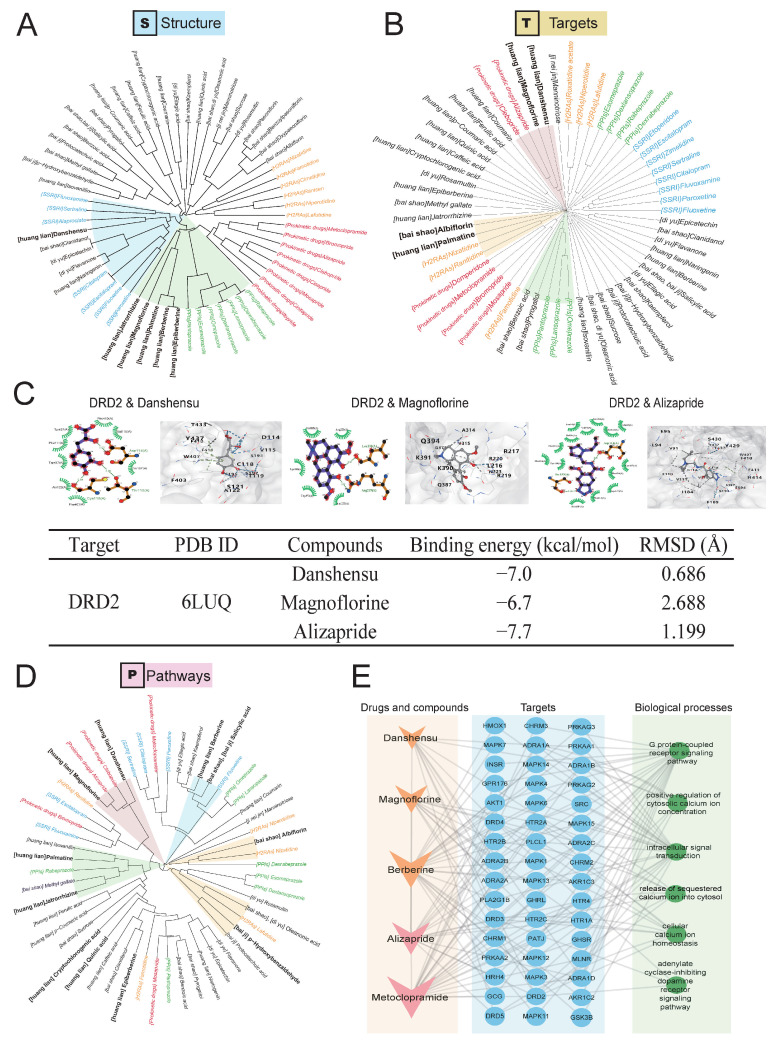
Identification of active compounds in QWZT based on a structure–target–pathway similarity algorithm. (**A**,**B**) Hierarchical clustering of QWZT compounds and FDA-approved drugs based on (**A**) chemical structure similarity and (**B**) target space similarity. Colors denote: black (QWZT compounds), red (prokinetic drugs), blue (SSRIs), green (PPIs), orange (H2RAs). Similar QWZT compounds (maximum peak area ratio > 1%) are highlighted in bold black text. (**C**) Molecular docking results of the DRD2 receptor with danshensu, magnoflorine, and alizapride. (**D**) Hierarchical clustering based on pathway similarity. (**E**) Hierarchical network of drugs, compounds, targets, and biological processes. Colors represent: orange (QWZT compounds), pink (drugs), blue (targets), green (biological processes). Node size for drugs/compounds corresponds to their degree centrality in the network.

Hierarchical clustering based on structural similarity revealed that danshensu, naringenin, flavanone, epicatechin, and cianidanol showed a similar structure to SSRIs. While magnoflorine, berberine, epiberberine, jatrorrhizine, and palmatine demonstrated closer structural alignment with A02B (Figure 3A). These findings suggest that some compounds in QWZT exhibited anti-acid and brain–gut regulation activities in chronic gastritis.

To further clarify the target similarity of QWZT compounds with FDA drugs, we performed target-based clustering. Results indicated that albiflorin and palmatine were similar to H2RAs. Pyrogallol and benzoic acid were similar to PPIs. Danshensu and magnoflorine demonstrated notable similarity to prokinetic drugs (Figure 3B). Prokinetic drugs suppress DRD2, a key protein in prokinetic drug mechanisms. Given the strong evidence of target similarity between magnoflorine, danshensu, and prokinetic drugs, we conducted molecular docking studies to evaluate drug binding interactions with DRD2 (Figure 3C). Molecular docking confirmed that these compounds showed comparable binding affinities to DRD2, with binding energies of ≤−5.0 kcal/mol. To assess the reliability of the docking results, we calculated the RMSD values using AutoDock Vina (v1.1.2) [13]. The RMSD values for the complexes formed by danshensu, magnoflorine, and alizapride with D2R2 were 0.686, 2.688, and 1.199 Å (Figure 3C), respectively. The results suggested that magnoflorine and danshensu showed potential activity in regulating gastrointestinal smooth muscle and that the conformations of their complexes with D2R2 were relatively stable.

Consistent with these observations, pathway similarity analysis revealed that most herbal compounds shared functional pathways with FDA-approved drugs (Figure 3D). Berberine and salicylic acid share pathways with fluoxetine (SSRIs), albiflorin, oleanonic acid, p-Hydroxybenzaldehyde share pathways with H2RAs. Palmatine and jatrorrhizine share pathways with PPIs.

QWZT compounds exhibited similarities with prokinetic drugs in multidimensional evidence (structure, target, and pathway). Hence, we constructed a network to visualize the associations between prokinetic drugs, herbal compounds, biological processes, and the involved targets. Prokinetic drugs mainly exert their effects by acting on the gastrointestinal smooth muscle, while calcium ion concentration, G protein-coupled receptor signaling pathways, and dopamine receptor signaling pathways can regulate contraction or relaxation in gastrointestinal smooth muscle. The results (Figure 3E) showed that berberine and magnoflorine may be the key compounds in the network, suggesting that they have a potential efficacy similar to regulating gastrointestinal smooth muscle.

### 2.4. Prediction and Validation of the Potential Regulatory Effects of QWZT Compounds with Similarities to FDA-Approved Drugs on Gastrointestinal Smooth Muscle

**Figure 4:**
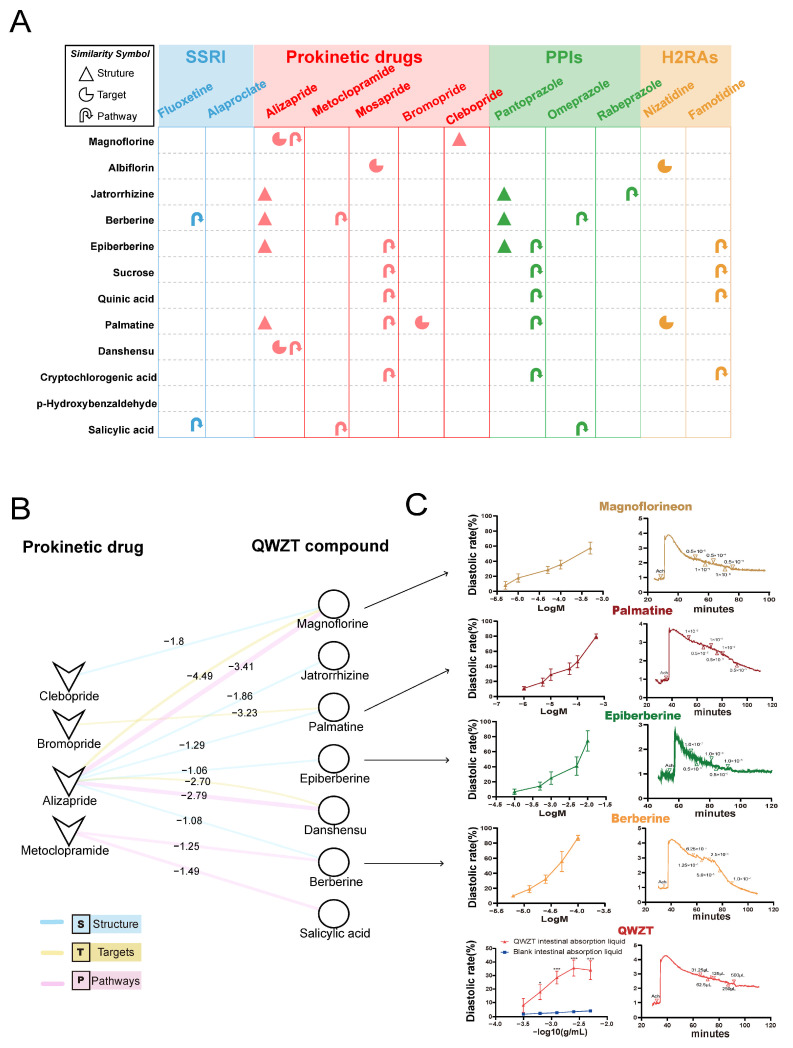
Discovery and validation of potential pharmacological activities of QWZT compounds based on similarity with clinical approved drugs. (**A**) Similarity table between QWZT compounds and FDA-approved drugs. (**B**) QWZT compound–prokinetic drug similarity network based on structure–target–pathway similarity. Blue, yellow, and pink represent structure, target, and pathway similarity. Thicker edge reflects greater similarity. The numbers in the figure represent z-scores. (**C**) Gastrointestinal smooth muscle antispasmodic experiment. Acetylcholine-induced contraction of the gastrointestinal smooth muscle, QWZT intestinal absorption liquid (*n* = 6), blank intestinal absorption liquid (*n* = 3), berberine, epiberberine, palmatine, and magnoflorine (*n* = 6). Data are expressed as the mean ± SD, * *p* < 0.05, *** *p* < 0.001.

Similar compounds were summarized in Table S2. In terms of the number of QWZT compounds similar to FDA-approved drugs, we found 21/33 (63%) compounds were similar to prokinetic drugs. They accounted for the largest proportion in terms of quantity. To comprehensively predict pharmacological activity of QWZT compounds, we concluded the most similar FDA-approved drugs for each QWZT compound (maximum peak area ratio > 1%) based on similarity of structure, targets and pathway fingerprints (Figure 4A). Only two compounds were predicted to have SSRIs activities (berberine, salicylic acid). A total of 11 out of 12 compounds have a similar structure, target or pathways to prokinetic drugs. Moreover, 9/12 compounds were predicted to have anti-acid activity (similar to A02B).

Prokinetic drugs were identified as the FDA-approved drugs most similar to QWZT compounds, supported by multidimensional evidence (structural similarity, target alignment, and pathway involvement). To prioritize compounds with high similarity to prokinetic drugs, we selected those with a maximum peak area ratio > 1% and z-score < −1 (Tables S2 and S3). Using these criteria, we constructed a network linking QWZT compounds to prokinetic drugs based on similarities in chemical structure, target interactions, and biological pathways (Figure 4B). Among them, magnoflorine and danshensu exhibited the highest pathway similarity (z-score: −3.41, −2.79) to alizapride. Notably, berberine, palmatine, and magnoflorine demonstrated similarity to two prokinetic drugs. Magnoflorine stood out as the predominant constituent in QWZT, showing multidimensional similarity to prokinetic drugs across structure, target, and pathway dimensions.

To explore potential regulatory effects on gastrointestinal smooth muscle of QWZT compounds in (Figure 4B), we performed a literature review of the top five compounds by maximum peak area ratio (Table 2). Jatrorrhizine and berberine were documented to directly exert effects on gastrointestinal smooth muscle. In contrast, magnoflorine, epiberberine, and palmatine lacked direct experimental evidence for this effect.

To explore the relaxation effects on gastrointestinal smooth muscle of novel compounds without literature evidence (magnoflorine, epiberberine, and palmatine), antispasmodic experiments for gastrointestinal smooth muscle were applied (Figure 4C). Berberine was chosen as the positive control for subsequent experiments due to its well-documented effects. Magnoflorine, epiberberine, and palmatine were prioritized for further investigation into their potential smooth muscle relaxant activities. Epiberberine, palmatine, and magnoflorine were capable of relaxing acetylcholine-induced gastrointestinal smooth muscle contraction in a concentration-dependent manner. Berberine, epiberberine, and palmatine achieved maximum relaxation rates exceeding 80%, while magnoflorine reached approximately 60%. These results suggest that these compounds contribute significantly to QWZT’s regulatory effects on gastrointestinal smooth muscle. In the antispasmodic assay (Figure 4C), QWZT’s intestinal absorption solution induced a concentration-dependent relaxation of gastrointestinal smooth muscle contraction caused by acetylcholine, achieving a maximum relaxation rate of 40%. Compared to the blank intestinal absorption solution at the same dilution, the effect was statistically significant, which implies that QWZT showed prominent anti-contraction effects on smooth muscle. These results suggest that they may exert a relaxant effect by antagonizing the muscarinic receptor agonist acetylcholine.

**Figure 5:**
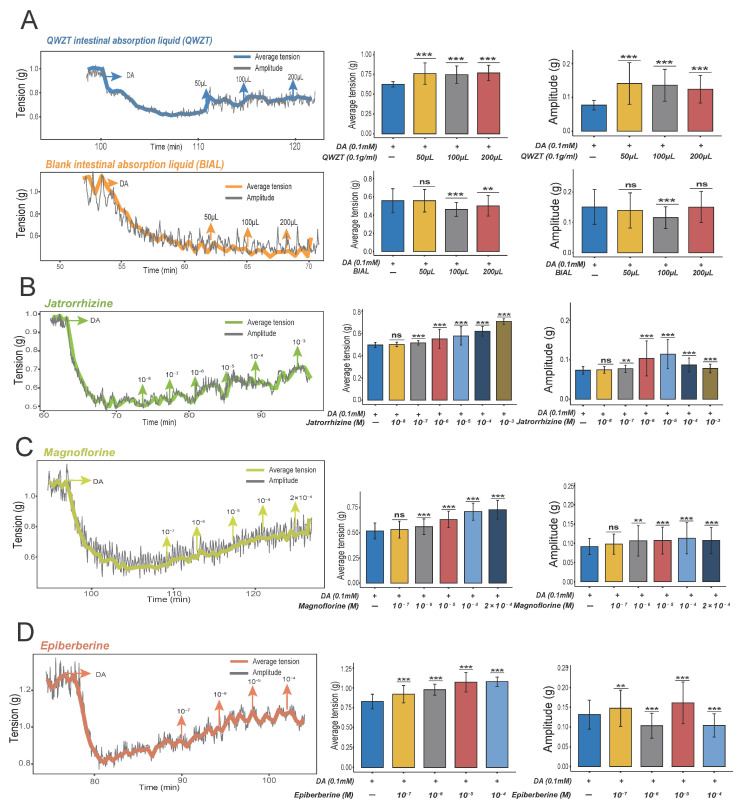
Validation of the inhibitory effects of QWZT and its similar compounds on the relaxation of isolated rat duodenum induced by the DRD2 receptor agonist dopamine. The effects of QWZT (*n* = 6) and blank intestinal absorption liquid (*n* = 3) (**A**), jatrorrhizine (*n* = 6) (**B**), magnoflorine (*n* = 6) (**C**), and epiberberine (*n* = 6) (**D**) on average tension and amplitude. Data were expressed as the mean ± SD, *p* > 0.05 (ns), ** *p* < 0.01, *** *p* < 0.001 vs. dopamine group.

To explore the contraction effects on gastrointestinal smooth muscle, the DRD2 agonist dopamine was used to induce intestinal relaxation. Magnoflorine has already demonstrated excellent binding affinity for the DRD2 receptor (Figure 3C), while the contractile ability of jatrorrhizine has been reported in the literature (Table 2). Epiberberine, with its higher peak area and weaker relaxing ability (Figure 4C, Table 2), was also selected to verify the contraction effect. The results indicate that QWZT can significantly increase the average tension and amplitude in a concentration-dependent manner, while the blank intestinal absorption fluid cannot increase the average tension and amplitude (Figure 5A). Similarly, jatrorrhizine and magnoflorine can also significantly increase the average tension and amplitude (Figure 5B,C). Epiberberine can significantly increase the average tension, but its effect on amplitude was fluctuating (Figure 5D). These results suggest that they may exert a contractile effect by antagonizing the DRD2 receptor agonist dopamine.

### 2.5. QWZT-Driven Multi-Target Network Perturbation of Undruggable Pathology in Gastritis-Related Diseases

The key pathological processes in chronic gastritis—mucosal injury, inflammation, and immune dysregulation—lack direct therapeutic interventions, unlike symptomatic treatments such as prokinetics, anti-acids, or SSRIs. To investigate the effect of QWZT on these undruggable processes, we evaluated how perturbation of QWZT compound targets impacts the robustness of associated disease networks.

**Figure 6:**
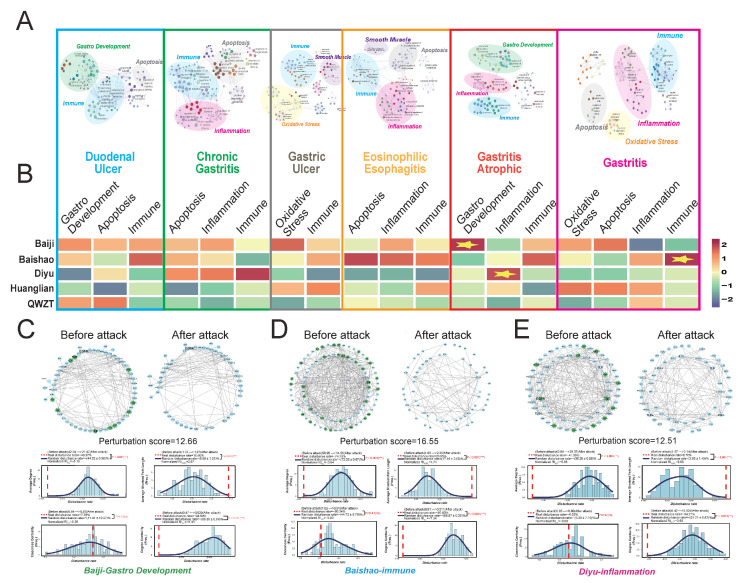
QWZT effect on undruggable pathology in gastritis-related diseases based on multi-target perturbation algorithm. (**A**) Construction of a holistic network and specific functional subnetworks targeting six diseases related to gastritis. Each color represents a distinct functional subnetwork, with grouped GO terms sharing the same color (blue represents immune, pink represents inflammation, yellow represents oxidative stress, gray represents apoptosis, green represents gastro development, and purple represents smooth muscle). Node size corresponds to the number of genes associated with each GO term. (**B**) Heatmap of perturbation score for herbal medicines in the disease networks. The stars in the figure represent the highest scores for each herb and its pathological processes. (**C**–**E**) The target perturbation effect of herbal ingredients on disease network and the distribution of its four topological features in real network. The green nodes represent the herbal ingredients’ targets. The size of the labels in the network is proportional to the degree of the node. Data are expressed as the mean ± SD, *p* > 0.05 (ns), ** *p* < 0.01, *** *p* < 0.001.

First, protein–protein interaction (PPI) networks were constructed for six gastritis-related diseases (Figure S1). Subnetworks corresponding to core pathological processes were identified through gene functional enrichment analysis. Among these, proteins about smooth muscle contraction were targeted by prokinetic and SSRIs drugs. But pathological processes about oxidative stress, inflammation, immune response, and gastro development were undruggable processes without directly targeting FDA-approved drugs (Figure 6A).

Using a multi-target perturbation algorithm, we quantitatively assessed each herb’s perturbation score (combining disturbance rate of Average Degree-AD, Average Shortest Path Length-ASPL, Closeness Centrality-CC, and Degree Centrality-DC) to interfere with undruggable pathological processes. Heatmap of perturbation score to each undruggable process shows that Baiji primarily affected the gastro development subnetwork, Diyu perturbed the inflammation, and Baishao targeted the immune subnetwork (Figure 6B).

The impact of deleting drug targets on network robustness was evaluated by analyzing changes in topological features (AD, ASPL, CC, and DC). As examples, all four topological features were displayed for disturbance to gastro development subnet of Baiji (Figure 6C), disturbance to immune subnet of Baishao (Figure 6D), and disturbance to inflammation subnet of Diyu (Figure 6E). Baiji’s disturbance to gastro development subnet reduced the subnetwork from 221 edges to 112 and 60 nodes to 50, significantly altering AD, ASPL, and DC (Figure 6C). The disturbance rate of AD (−49.37%) substantially exceeded random attack effects (*p* < 0.001), demonstrating Baiji’s strong destabilizing effect on this subnetwork. Similarly, Baishao’s target perturbation of immune subnetwork reduced edges from 367 to 69 and nodes from 99 to 66, significantly altering AD, ASPL, and DC (Figure 6D) compared with random attack effects (*p* < 0.001). Diyu targeting inflammation subnet reduced edges from 253 to 121 and nodes from 71 to 58 (Figure 6E). Different from FDA-approved drugs, QWZT had treatment potential on undruggable processes of CG (inflammation, immune, and gastro development).

### 2.6. Discovery and Validation of QWZT and Its Specific Compounds Activities on Undruggable Process

**Figure 7:**
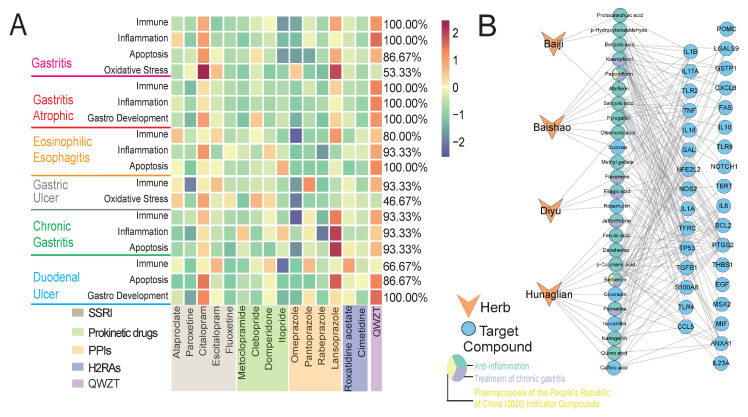
Discovery of specific QWZT compounds compared to FDA-approved drugs. (**A**) Heatmap of perturbation score for QWZT and FDA-approved drugs. The ratio indicates the proportion of instances where the perturbation score of QWZT surpasses that of FDA-approved drugs within the same pathological process. (**B**) “Herb-compound-target” network of anti-inflammation compounds of QWZT. Orange nodes represent herbs of QWZT, while blue nodes indicate targets in the inflammation subnetwork of chronic gastritis. Literature mining of anti-inflammatory compounds in QWZT based on three types of evidence (yellow, green, and purple).

Leveraging a multi-target perturbation algorithm, we quantitatively assessed the impact of QWZT on gastritis disease networks relative to FDA-approved drugs to highlight the advantageous targeting of pathological processes of QWZT. QWZT induced significantly greater network disruption in chronic gastritis and atrophic gastritis disease networks, surpassing the perturbation magnitude of 93.33% and 100% of FDA-approved drugs, respectively (Figure 7A). At the pathological process level, QWZT exerted stronger perturbations in undruggable subnets (immune, apoptosis, inflammation) than FDA-approved drugs. The immune subnetwork (disrupted by QWZT across all six diseases, exceeding 88.89% of FDA drugs) and inflammation subnetwork (disrupted by QWZT in four diseases, exceeding 96.67% of FDA drugs) emerged as QWZT’s primary mechanistic foci. This perturbation profile, consisting of GO enrichment results implicating inflammation regulation, supports our focused investigation QWZT mechanism for the inflammation pathological process.

To identify QWZT compounds involved in the inflammation pathological process, we constructed the “herb-compound-target” network (Figure 7B and Figure S4A). There were 25 compounds targeting proteins in inflammation subnetworks and being identified as potential anti-inflammatory compounds of QWZT (Table S4). We conducted literature mining on these compounds (Table S5) and selected candidates for subsequent validation based on three types of evidence. Finally, 19 compounds have been reported to possess anti-inflammatory activity, with seven of them not only exhibiting anti-inflammatory effects but also demonstrating therapeutic efficacy against chronic gastritis. As indicator compounds, berberine in Huanglian and paeoniflorin in Baishao were selected to be representative anti-inflammation compounds for experimental validation.

**Figure 8:**
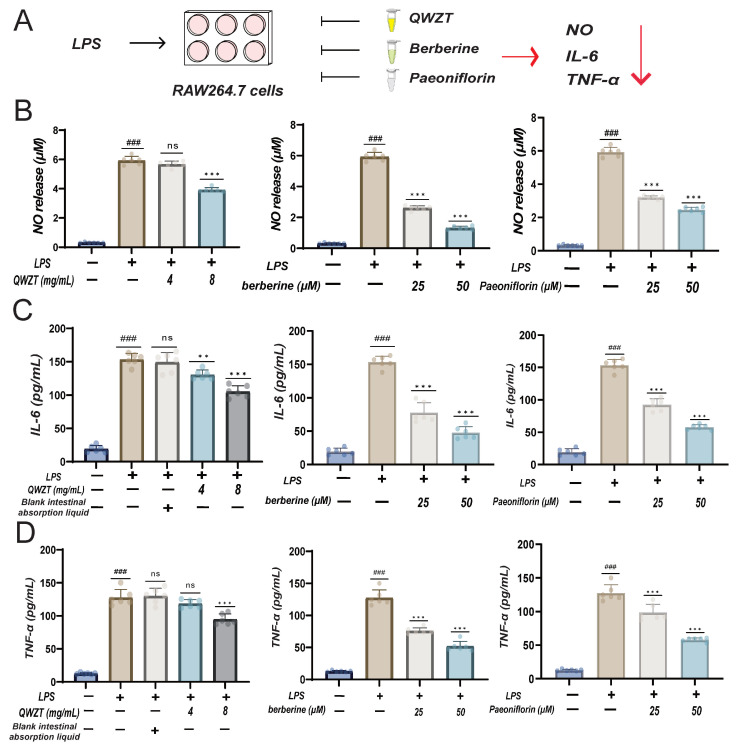
Validation of the anti-inflammatory activity of QWZT and its specific compounds in the LPS-induced RAW264.7 cells inflammation model. (**A**) The workflow of RAW264.7 cells experiment. The arrow on the right of LPS represents the creation of an inflammatory environment in cells upon LPS addition. The arrow on the left of the drug represents the drug’s inhibitory effect on LPS induced responses. The red arrow indicates the final result after drug addition: a decrease in inflammatory factor levels. Effects of QWZT, berberine, and paeoniflorin on the expression of NO (*n* = 6) (**B**), IL-6 (*n* = 6) (**C**), and TNF-α (*n* = 6) (**D**). Data were expressed as the mean ± SD, ^###^ *p* < 0.001 vs. control group, *p* > 0.05 (ns), ** *p* < 0.01, *** *p* < 0.001 vs. LPS group.

The LPS-induced inflammatory model in RAW264.7 and GES-1 cells was used to validate the anti-inflammatory effects of QWZT-specific compounds. First, we evaluated the anti-inflammatory capacity of the LPS-induced RAW264.7 cells inflammation model. We found that 8 mg/mL QWZT, berberine, and paeoniflorin (25 and 50 μM) all significantly reduced the concentration of NO, while 4 mg/mL of QWZT demonstrated a trend of reducing NO levels; this effect did not reach statistical significance (Figure 8B). ELISA was performed to assess the expression of IL-6 and TNF-α. The results revealed that QWZT, berberine, and paeoniflorin significantly suppressed the expression of both IL-6 and TNF-α (Figure 8C,D). However, 4 mg/mL of QWZT only significantly reduced IL-6 expression, while its effect on TNF-α expression was not statistically significant. Notably, at the same concentration, berberine exhibited stronger anti-inflammatory effects than paeoniflorin, a finding also further confirmed in subsequent experiments.

**Figure 9:**
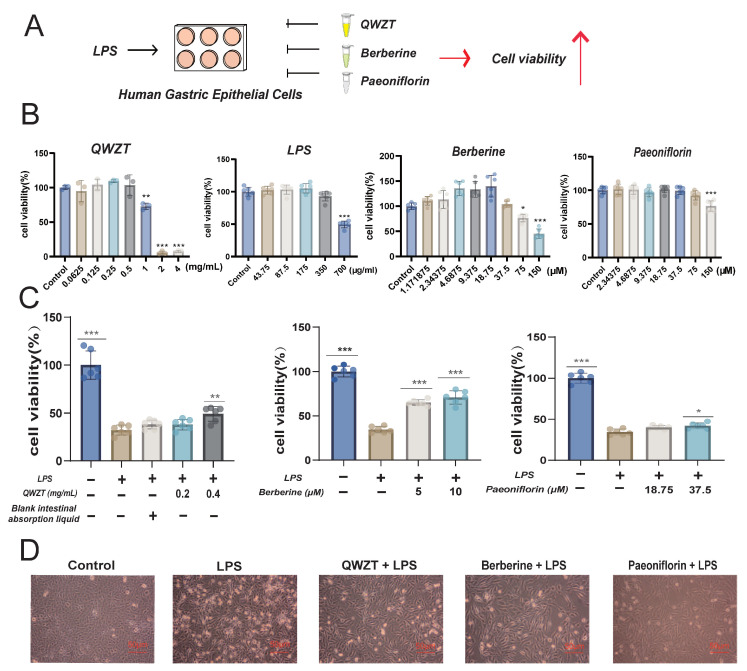
Validation of the anti-inflammatory activity of QWZT and its specific compounds in the LPS-induced GES-1 cells inflammation model. (**A**) The workflow of GES-1 cells experiments. The arrow on the right of LPS represents the creation of an inflammatory environment in cells upon LPS addition. The arrow on the left of the drug represents the drug’s inhibitory effect on LPS-induced responses. The red arrow indicates the final result after drug addition: enhanced cell viability. (**B**) Assessment of the appropriate concentrations of the drug, QWZT (*n* = 3), LPS, berberine, and paeoniflorin (*n* = 6). (**C**) Evaluation of the QWZT, berberine, and paeoniflorin (*n* = 6) protective effects. (**D**) Morphological changes in GES-1 cells in response to drug treatments. Data were expressed as the mean ± SD, * *p*< 0.05, ** *p* < 0.01, *** *p* < 0.001 vs. LPS group.

In the LPS-induced GES-1 cells inflammation model, we assessed the appropriate concentrations of drug. We found that QWZT ≥ 1 mg/mL, berberine ≥ 37.5 μM, and paeoniflorin ≥ 150 μM would significantly reduce cell viability (Figure 9B). 700 μg/mL LPS was able to significantly decrease cell viability, which was considered an appropriate concentration to cause acute GES-1 cell damage and inflammation. As expected, QWZT and its specific compounds can significantly protect damaged GES-1 cells. Specifically, QWZT can increase cell viability from 32% to 49%, berberine from 34% to 70%, and paeoniflorin from 34% to 38% (Figure 9C). Morphological changes revealed distinct differences among the groups (Figure 9D). In the control group, cells exhibited a fusiform shape with a high cell density and few floating cells. In contrast, the QWZT, berberine, and paeoniflorin groups showed an increased number of floating dead cells, with the remaining cells losing their original epithelial-like morphology. They transformed into irregularly elongated fusiform shapes and displayed the formation of pseudopodia. However, the most dramatic morphological alterations were observed in the LPS group, which was marked by significant cell death.

## 3. Discussion

CG involves multifactorial pathogenesis [20,21]; for instance, *H. pylori* infection, inflammation, autoimmune responses, and smooth muscle dysfunction. TCM offers distinct advantages for such complex diseases through inherent multi-pharmacological actions [22]. However, the multi-component and multi-target characteristics of TCM make the elucidation of its mechanisms a challenging problem [23]. In this study, we established a dual-algorithm integration framework to investigate both druggable processes (where QWZT shares mechanisms with FDA-approved drugs) and undruggable processes (where QWZT exhibits unique targeting capabilities). This framework effectively elucidates the potential mechanisms of QWZT in the treatment of CG and can be extended to other multi-component drugs.

Traditional Chinese Medicine-Network Pharmacology (TCM-NP) emerged as the result of cross-innovation between traditional medicine, modern pharmacology, information science, and systems science [24]. The concepts central to TCM-NP precede the introduction of “network pharmacology” in 2007 [25]. TCM-NP methodologies have been successfully applied to the selection of appropriate medications for the treatment of gastritis [26]. However, it cannot quantitatively evaluate the advantages of herbs on specific pathological processes of diseases, nor can it effectively compare the varying degrees of efficacy among different herbs. The application of the perturbation algorithm can address this issue to some extent, thereby identifying the pathological processes through which the herbal medicine exerts its therapeutic advantages. It should be emphasized that this “advantage” is measured based on the perturbation strength of the herbal medicine on the disease pathological subnetwork. The algorithm is versatile and has been applied in hypertension, nephropathy, and COVID-19 drug discovery [27,28]. Likewise, the algorithms can be readily extended to other TCM formulas.

TCM contains numerous compounds, and to systematically elucidate its therapeutic mechanisms, it is essential to clarify the specific roles of each compound in disease. In contrast, Western medicines are typically characterized by single-compound composition and well-defined mechanisms. Comparing TCM compounds with Western drugs represents an effective research strategy. However, experimentally screening dozens or even hundreds of TCM compounds one by one is undoubtedly time-consuming and labor-intensive. By employing the structure–target–pathway similarity algorithm, we have successfully identified TCM compounds that share similarities with Western drugs. This approach enables a preliminary, comprehensive analysis of the therapeutic mechanisms underlying TCM’s multi-compound system. The algorithm has been applied to XiaoErFuPi granules and identified vanillic acid, which was similar to cimetidine [29]. Similarly, the algorithms could also be applied to other TCM formulas.

Phytochemical profiles of the same botanical species can differ substantially among cultivation regions, harvest seasons, and extraction protocols, leading to variable in vivo bioavailability and therapeutic potency [30]. The bioactive compounds in QWZT undoubtedly exhibit certain batch-to-batch variations. In our experiments, we will utilize raw materials from the same batch that met pharmacopeial standards. In our future work, we will perform absolute quantification of the main compounds and determine their LOD and LOQ. Furthermore, we strongly advocate that pharmaceutical manufacturers enhance standardized cultivation and strictly adhere to Good Manufacturing Practice (GMP) processing protocols, thereby minimizing therapeutic variability arising from herbal batch differences.

With the popularization of 16S rRNA gene sequencing and high-throughput metagenomic technologies, we have gradually recognized the close association between gut microbiota and diseases, and CG is no exception. The gut microbiota plays a critical role in maintaining gastrointestinal homeostasis, immune regulation, and metabolic processes [31]. In gastric cancer, the gut microbiome loses diversity and becomes enriched in pro-inflammatory, carcinogenic bacteria [32]. Oral herbal medicine reshapes the gut microbiota, which in turn can be involved in the therapeutic process of the disease [33]. For instance, QWZT can increase the abundance of beneficial bacteria and decrease the abundance of detrimental bacteria, and after fecal microbiota transplantation, it effectively improved acetic acid-induced gastric ulcers [11].

Admittedly, there were certain limitations in this study. The complex molecular mechanisms underlying QWZT were not fully explored. Some drugs (antibiotics, antacids, and gastric mucosal protectors) commonly used in triple or quadruple therapies were excluded in perturbation analysis, meaning that they were not included as potential comparisons for QWZT. Antibiotics function through bactericidal targets, whereas antacids and gastric mucosal protectors (ATC code: A02BX, for instance, troxipide, and pirenzepine) primarily operate via chemical neutralization or physical barrier formation rather than acting on the specific gastric targets. Without a gastric target, antibiotics and gastric mucosal protectors cannot be included in our perturbation algorithm. Consequently, we cannot treat the triple/quadruple combination as an integral unit for comparison. To address this limitation, we will incorporate these combination therapies as positive controls in future in vivo studies, enabling empirical comparison of their overall efficacy relative to QWZT. The therapeutic effects of QWZT and its compounds have not yet been validated in vivo for chronic gastritis, which is currently a key limitation of this study. It is imperative to address these limitations by conducting further in vivo and in vitro experiments.

In our future research plan, we intend to employ an ethanol–indomethacin combination-induced rat model of CG to address the current lack of in vivo validation in our study. This model is well-established and reliably replicates key pathological features of human CG, including gastric mucosal injury, inflammatory infiltration, and gastric motility dysfunction [34,35]. Our algorithm has revealed the anti-inflammatory activity of QWZT and its regulatory effects on gastrointestinal smooth muscle. Therefore, in terms of anti-inflammatory activity, hematoxylin–eosin staining of gastric sections will be performed to score the degree of inflammatory infiltration. ELISA will be used to quantify pro-inflammatory cytokines in both serum and gastric tissue, while Western blot and immunohistochemistry will assess the protein expression of inflammation-related markers in gastric tissue. These evaluations aim to demonstrate the anti-inflammatory activity of QWZT. Regarding the regulation of gastrointestinal smooth muscle, c-Kit immunohistochemistry will be carried out to enumerate interstitial cells of Cajal (ICC) in the submucosal layer, and serum levels of motilin, gastrin, and substance P will be measured to evaluate QWZT’s regulatory effects on gastrointestinal smooth muscle. The doses were selected based on the clinically equivalent dose and published reference [11]. First-line triple/quadruple eradication regimens will serve as the positive control. Regarding safety, an acute toxicity study will first be conducted to determine the maximum tolerated dose. During the observation period, body weight, food intake, general behavior, and mortality will be recorded daily, and on the final day, serum biochemical parameters reflecting liver and kidney function markers will be measured. We believe that subsequent refinement of this experiment will provide a more solid foundation for elucidating the mechanism of QWZT in treating CG.

## 4. Materials and Methods

### 4.1. Preparation and Identification of Intestinal Absorption Fluid of QWZT

#### 4.1.1. Preparation of the QWZT Everted Gut Sac Liquid

To prepare the Tyrode buffer, a solution was prepared by dissolving 8.0 g NaCl, 1.0 g NaHCO_3_, 0.28 g KCl, 0.1 g MgCl_2_, and 0.05 g NaH_2_PO_4_ in 500 mL of distilled water. The mixture was sealed and refrigerated. Concurrently, 0.2 g CaCl_2_ was dissolved in another 500 mL of distilled water and stored under the same conditions. For utilization, the two solutions were evenly mixed, and 1.0 g of glucose was added to complete the preparation. 120 g QWZT powder was dissolved in 600 mL Tyrode buffer to create a saturated solution with a concentration of 0.2 g/mL.

Adult male Sprague–Dawley rats weighing 230–250 g were humanely euthanized via cervical dislocation after fasting for at least 12 h. The intestine was quickly dissected into four segments (duodenum, jejunum, ileum, and colon). Each segment was washed with cold Tyrode buffer, tied with a silk thread, filled with 2 mL Tyrode buffer, and firmly tied at the other end. The sac was transferred to a Magnus bath containing 20 mL QWZT liquid with oxygenated media (95% O_2_/5% CO_2_) at 37 °C. After 2 h, the samples underwent preservation through a 0.22 μm microporous filtration process to ensure sterility and sample integrity [36,37].

#### 4.1.2. Identification of Intestinal Absorbable Compounds of QWZT by UPLC-MS

QWZT intestinal absorbable compounds were analyzed by the UHPLC-Q-Orbitrap HRMS system of Ultimate 3000 HPLC (Dionex, Sunnyvale, CA, USA) and Thermo Q Exactive Plus HRMS (Thermo Fisher Scientific, Waltham, MA, USA). For the chromatographic separation, a column of Waters ACQUITY UPLC HSST3 (2.1 mm × 100 mm, 1.8 μm; Waters Corporation, Milford, MA, USA) was used at a temperature of 35 °C and a flow rate of 0.2 mL·min^−1^. The mobile phase consisted of 0.1% formic acid in acetonitrile (a) and 0.1% formic acid in water (b), with a gradient elution as follows: 0–10 min, 100% B; 10–20 min, 100–70% B; 20–25 min, 70–60% B; 25–30 min, 60–50% B; 30–40 min, 50–30% B; 40–45 min, 30–0% B; 45–60 min, 0% B; 60–60.1 min, 0–100% B; 60.1–70 min, 100% B. The DAD wavelength was set to sweep from 190 nm to 400 nm.

In terms of mass spectrometry, this study employed positive and negative ion modes with a heated electrospray ionization (HESI) source for mass detection. The positive spray voltage was set at 3.2 kV, while the negative was 3.0 kV, achieving a resolution of 70,000 MS and 17,500 MS/MS. The mass scanning range for both modes extended from *m*/*z* 100 to *m*/*z* 1500. Quasimolecular ion peaks, fragment ion data, and retention times were analyzed and compared between the QWZT and blank groups using the UHPLC-Q-Orbitrap system. Unknown compounds were identified via Compound Discover 3.2 software, which utilized the McCloud and MzVault databases for peak matching, alignment, noise filtering, and normalization. The compounds identified in both the everted gut sac liquid and QWZT powder are the intestinal absorbable compounds of QWZT.

### 4.2. Prediction and Functional Enrichments of Targets in Intestinal Absorbable Compounds and FDA-Approved Drugs

#### 4.2.1. Targets Prediction of Intestinal Absorbable Compounds

The molecular structure of intestinal absorbable compounds was downloaded from the ChemSpider database. Potential targets were obtained through BATMAN-TCM [38], STITCH, and the PubchemBioassay database. Targets with high confidence (combined score greater than 0.2) were selected as potential drug targets. The Venn diagram was developed using the OECloud tools at (https://cloud.oebiotech.com (accessed on 28 June 2024)).

#### 4.2.2. Targets Prediction of FDA-Approved Drugs

A02B is commonly used for the treatment of peptic ulcers and gastroesophageal reflux disease. Among A02B (A02BA, A02BC, and A02BX), A02BX works through physical and chemical mechanisms to treat gastritis rather than directly targeting a specific protein [39]. Therefore, A02BX was not included in the subsequent analysis. A02BA and A02BC (PPIs and H2RAs) were treated as a whole for GO analysis. Targets of FDA-approved drugs can be obtained through the LINCS [40]. We utilized the L1000 gene expression dataset to examine gene expression profiles and the transcriptomic changes in 10 selected cell types after 6 and 24 h of drug treatment. Pearson correlation analysis was performed to identify gene expression changes significantly associated with drug dosage. After integrating and analyzing, the top 500 genes with correlation coefficients were defined as potential (direct or indirect) targets of FDA-approved drugs [41,42].

#### 4.2.3. GO Biological Process Enrichment Analysis of Drug Targets

We utilized Metascape [43] to perform Gene Ontology (GO) enrichment analysis for the targets of QWZT, FDA-approved drugs, and their common targets under the screening condition of *p* < 0.01.

### 4.3. Structure–Target–Pathway Similarity Algorithm

#### 4.3.1. Drug Similarity Evaluation Based on Meta Path Searching in “Compound-Target-Pathway” Heterogeneous Network

The “compounds-target-pathway” network integrates three node types—compounds, targets, and pathways—linked by compound-target and target-pathway interactions. The BATMAN-TCM [38] algorithm predicted the former, while the latter was sourced from gene annotations in GO [44], Reactome [45], and KEGG [46], with GOA last updated in August 2021 and the others accessed in June 2021. Detailed principles regarding the algorithm can be found in our previous research [29].

FP2 fingerprint and functional group [42,47] were used to measure the chemical structure similarity. Molecular fingerprints represent molecular structures using a sequence of binary digits (bits) that indicate the presence or absence of specific substructures within a molecule. By comparing these fingerprints, we can assess the similarity between two molecules. After converting SMILES strings into scalar fingerprints, the Tanimoto coefficient was employed as the similarity score to quantify the degree of similarity between the two molecules [41].

As for target similarity, three kinds of targets, respectively, from STITCH, Pubchem, and BATMAN-TCM, were used to measure drug–target interactions. The combined score was derived by integrating the probabilities from three different sources of evidence, which were calculated as described below:

$$Combined\;Score=(1-P_{stitch})\times (1-P_{bioassay})\times (1-P_{batman})$$

P_stitch_, P_bioassay_, and P_batman_ were individually represented the probabilities of drug–target interaction from the literature mining resource STITCH, the compound bioactivity resource Pubchem Bioassay, and the prediction resource BATMAN-TCM. Only targets with a combined score greater than 0.4 were considered as potential targets in compounds.

The potential targets of compounds were used to measure the target similarity between two drugs. For instance, T_a_ represented the target space of drug a, and T_b_ represented the target space of drug b. The similarity score of S_a,b_ was as follows:

$$S_{a,b}=\frac{T_{a}\cap T_{b}}{T_{a}\cup T_{b}}$$

Pathway signature similarity of compounds was assessed using PathSim based on a “compound-target-pathway” heterogeneous network, a method originally from social network recommendation systems [48]. Pathsim has been adapted to uncover similar entities that share a common metapath within the heterogeneous network (exemplified by identifying compounds that exhibit similar pathway profiles). The similarity score of two drugs was calculated based on only 50 targets for each drug (randomly picked from the target space of the corresponding drug). One hundred similarity scores were generated after 100 random picks, and the ultimate similarity score of two drugs was the average score. Given a set of N compounds to be clustered, an N × N similarity matrix was generated (Table S1). Subsequently, utilizing the R package hClust (v1.2.6), we executed a hierarchical clustering analysis on the N compounds, grounded on the intricate similarity matrix derived from their pathway fingerprint comparisons. Moreover, we conducted quality control on the data, excluding drugs or compounds that were not correctly categorized (Figure S2). This approach clarifies how the compounds are related and pinpoints clusters that likely work through the same biological mechanism or have similar therapeutic potentials [49].

#### 4.3.2. Molecular Docking

The detailed information of the FDA-approved drug alizapride can be obtained from DrugBank (accession number DB01425). The structure of DRD2 was obtained from the RCSB Protein Data Bank database (https://www.rcsb.org/ (accessed on 6 June 2025)), accession code 6LUQ, while the structures of danshensu and magnoflorine were obtained from PubChem (https://pubchem.ncbi.nlm.nih.gov/ (accessed on 6 June 2025)), PubChem CID (11600642, 73337). Molecular docking simulations were performed using CB-Dock2tool [50], accessible via (https://cadd.labshare.cn/cb-dock2/php/index.php (accessed on 6 June 2025)). The 3D and 2D visualizations were completed by CB-Dock2tool and Ligplot+, respectively.

#### 4.3.3. Construction of Drug–Compound–Target–Biological Process and Similar Compound–Drug Network

Compounds were considered similar only if their similarity distance was below the first Q1 (Table S1); the distances will be multiplied by 100 for better comparison (Table S2). To select the most similar compounds, we focused on compounds with a maximum peak area ratio greater than 1%. Distance and similarity were inversely proportional. Using Z-scores to normalize the data, only Z-scores below −1 (Table S3) were included in the network. The network visualization was performed using Cytoscape (v3.9.1).

### 4.4. Experiments on the Regulatory Effects of Drugs on Gastrointestinal Smooth Muscle

#### 4.4.1. Animals

SPF male SD rats weighing 230–250 g were provided from Beijing Hua Fu Kang Biotechnology Co., Ltd., Beijing, China (Certificate NO. SCXK (Jing) 2019-0008). The experiment was approved by the Committee on Animal Care and Use of Beijing.

#### 4.4.2. Instruments and Reagents

Biopac MP150 Polychannel Physiological Recorder (Biopac, Goleta, CA, USA), Radnoti Ex vivo Tissue Perfusion System (Radnoti, Covina, CA, USA). Krebs–Henseleit (K-H) solution formulation: NaCl 118.96 mmol/L; KCl 4.73 mmol/L; KH_2_P0_4_ 1.17 mmol/L; MgSO_4_ 1.17 mmol/L; NaHCO_3_ 25.0 mmol/L; CaCl_2_ 2.54 mmol/L; Glucose 11.1 mmol/L. All reagents were of analytical grade.

#### 4.4.3. Description of the Isolated Intestinal Diastolic Assay

The experiment began by selecting rats that had fasted for 12 h but had free access to water. The animals were humanely euthanized via cervical dislocation, and a 10 cm segment of the proximal duodenum near the pylorus was immediately dissected and removed. The tissue was submerged in Krebs’s solution and meticulously cleaned by removing the mesentery and fat. The intestinal segment was then cut into approximately 1.5 cm lengths for subsequent use. The water bath temperature was maintained at 37 ± 0.5 °C. The Biopac MP150 physiological recorder was connected to a tension transducer, and one end of the intestinal segment was secured to an L-shaped aeration tube, fully submerged in 20 mL of Krebs’s solution. We gradually introduced a gas mixture consisting of 95% O_2_ and 5% CO_2_ into the setup. After a 15 min stabilization period, a 1 g weight was applied to the intestinal segment, and the experiment began once the contractions were steady. We ensured the nutrient solution was changed every 15 min to maintain optimal experimental conditions.

For the drug contraction experiment, dopamine (0.1 mM) was introduced to induce smooth muscle relaxation, and the drug was gradually added after the muscle relaxation reached a plateau. We divided the first 30 s after drug addition into 100 equal bins, computed amplitude and mean tension for each bin, and used these values for statistical comparisons. For the dopamine group, we analyzed the time points within the half-minute following the optimal relaxation effect induced by dopamine.

For the drug relaxation experiment, acetylcholine (0.1 μM) was introduced to induce smooth muscle contraction, and the drug was gradually added after the muscle contraction reached a plateau. The relaxation rate was calculated using the formula: Relaxation percentage (%) = (average tension before administration − average tension after administration)/average tension before administration × 100%.

### 4.5. Drug Disturbance on the Robustness of the Disease Network

#### 4.5.1. Construction of the PPI Network and Functional Subnetworks of Six Gastritis-Related Diseases

DisGeNET was used to search for disease genes [51], with the inclusion criterion being a Score gda ≥ 0.1. The filtered and deduplicated disease-related genes were imported into the STRING [52], and the human species was selected with a confidence level greater than 0.4. The PPI network was constructed based on the above-mentioned standards.

The GO enrichment analysis for disease genes was realized by Metascape under the screening condition of *p* < 0.01. The enriched terms were clustered into various subclusters using hierarchical clustering based on the similarity distance matrix of GO terms in Metascape. Based on the GO clustering network, GO terms with similar biological processes were merged into the same functional subnetwork.

#### 4.5.2. Multi-Target Perturbation Algorithm

After a drug interacts with the disease network, its robustness decreases, which can be used to predict the effect of multi-target drugs on the disease. Specifically, by removing drug targets from the disease network to simulate the treatment processes of the disease. Detailed principles regarding the algorithm can be found in our previous research [27,53]. In this study, the average shortest path length (ASPL), average degree (AD), degree centrality (DC), and closeness centrality (CC) served as four key topological features for evaluating the robustness of the network. The AD of a graph is simply the mean number of connections per node. The ASPL is one of the most robust indicators of network topology, which means that the average value of all the shortest path lengths in the network. The DC summarizes the overall centralization of a graph by aggregating the degree-based centrality scores of every node. The CC is another centralization measure for the whole graph, calculated from the closeness of all nodes.

The changes in the topological features of network robustness can be represented by the robustness index (RI), which was calculated as follows:

$$Robustness\;Index\;(RI)=\frac{{Topological\;Feature}_{after\;attack}-{Topological\;Feature}_{before\;attack}}{{Topological\;Feature}_{before\;attack}}$$

The normalized RI was calculated using the null distribution created from 100 random networks, providing a standard for comparing drug effects on the real disease network. The normalized RI was derived as the z-score of the real network’s RI relative to the null distribution, where $\bar{RI}$ represented the mean of the random RI, and S was the standard deviation of the random RI in the null distribution.

$$Normalized\;RI=\frac{{RI}_{real}-{\bar{RI}}_{random}}{S_{random}}$$

The RI of ASPL was negatively correlated with network robustness, while the AD, DC, and CC of RI were positively correlated with network robustness. The formula of the perturbation score was as follows:

$$Perturbation\;Score=Normalized\;{RI}_{ASPL}-Normalized\;{RI}_{AD}-Normalized\;{RI}_{DC}-Normalized\;{RI}_{CC}$$

### 4.6. Analysis of the Impact of QWZT and Its Herbs on Undruggable Pathology in Gastritis-Related Diseases

#### 4.6.1. Construction of the Network Before and After Drug Perturbation

The edges of targets were divided into compound-target and PPI interaction based on connections. Edges linking targets to compounds were defined as compound-target, while edges connecting targets to each other, generated via the STRING database, were defined as PPI interaction. We filtered the edges of the network. Edges of compound-target must have a combined score ≥ 0.2, while edges of PPI interaction need a combination score ≥ 0.9. We established the network before and after (drug target deletion) perturbation to intuitively simulate the disruptive effect of drugs on the disease network. The networks were constructed by Cytoscape (v3.9.1).

#### 4.6.2. Permutation Test of Real and Random Networks

The RI of four topological features (AD, ASPL, CC, and DC) was used to investigate whether there were significant differences in network robustness between a real network and a random network after the same drug attack. Null models were generated for the disease networks through randomization of the network with preserved node and edge numbers of the real network. A total of 100 random networks were generated as a benchmark, and 100 indicators were generated after the same drug attack on random networks as a null distribution for the permutation test [54].

### 4.7. Discovery Potential Anti-Inflammatory Activity of QWZT Compounds

The disease–inflammation–compound–target networks were constructed to discover potential anti-inflammation compounds. The functional inflammation subnetworks of gastritis, gastritis atrophic, chronic gastritis, and eosinophilic esophagitis were constructed by Cytoscape (v3.9.1). Edges of PPI interaction need a combination score ≥ 0.9.

We conducted literature mining for compounds involved in four inflammation networks based on three types of evidence (anti-inflammation, treatment of chronic gastritis, and pharmacopeia of the People’s Republic of China (2020) indicator compounds).

### 4.8. LPS-Induced Inflammatory Model in RAW264.7 Cells

#### 4.8.1. Cell Culture and Treatment

RAW264.7 cells were generously provided by Dr. Hu from the Institute of Chinese Materia Medica and cultured in DMEM (Gibco, New York, NY, USA) containing 10% FBS (Gibco, New York, NY, USA) and 1% penicillin and streptomycin (Gibco, New York, NY, USA) at 37 °C in a 5% CO_2_ humidified atmosphere. The medium was changed every two days, and cells were passaged when the density exceeded 80%.

#### 4.8.2. Solution Preparation

Berberine and paeoniflorin (Beijing Solarbio Science & Technology Co., Ltd., Beijing, China, purity greater than 98%) were dissolved in DMSO and treated by 0.22 μm microporous filtration to store at 0.1 mM.

The preparation of QWZT everted gut sac liquid was as mentioned in 4.1.1.

#### 4.8.3. Nitric Oxide (NO) Measurement

Cells planted in 24-well plates (1 × 10^5^/well) were preserved by paeoniflorin, berberine, and QWZT for 2 h, then incubated with LPS (1 μg/mL) for 16 h. NO release from the RAW264.7 cells was quantified with the Griess reagent (Beyotime, Shanghai, China).

#### 4.8.4. Enzyme-Linked Immunosorbent Assay (ELISA)

ELISA (Elabscience, Wuhan, China) was used to assess the expression of inflammatory cytokines, including IL-6 and TNF-α. All the experiments were conducted strictly following the manufacturer’s protocol.

### 4.9. LPS-Induced Inflammatory Model in GES-1 Cells

#### 4.9.1. GES-1 Cell Culture and Treatment

GES-1 cells were purchased from the MeisenCTCC (Jinhua, Zhejiang, China) and cultured in RMPI1640 (Gibco, USA) containing 10% FBS (Gibco, USA) and 1% penicillin and streptomycin (Gibco, USA) at 37 °C in a 5% CO_2_ humidified atmosphere. The medium was changed every two days, and cells were passaged when the density exceeded 80%.

#### 4.9.2. Cell Viability Assay

Cell viability was assessed using the Cell Counting Kit-8 (CCK-8, Dongren Chemical Technology Co., Ltd., Shanghai, China). Following the addition of 10 μL CCK-8 to each well, samples were incubated for 1–4 h. Absorbance was then measured at 450 nm using a microplate reader (Molecular Devices, San Jose, CA, USA).

#### 4.9.3. Evaluation of Toxicity of Drugs in GES-1 Cells

GES-1 cells were seeded in 96-well plates (8 × 10^3^/well) and cultured for 24 h in complete medium. The cells were, respectively, treated with QWZT intestinal absorption fluid at concentrations of 4, 2, 1, 0.5, 0.25, 0.125, and 0.0625 mg/mL; berberine at concentrations of 1.171815, 2.34375, 4.6875, 9.375, 18.75, 37.5, 75, 150 μM; paeoniflorin at concentrations of 2.34, 4.68, 9.37, 18.75, 37.5, 75, and 150 μM; and LPS (Beijing Solarbio Science & Technology Co., Ltd.) at concentrations of 43.75, 87.5, 175, 350, and 700 μg/mL. The cell viability was evaluated by CCK-8.

#### 4.9.4. Evaluation of Drug Effects

GES-1 cells were pretreated with QWZT (negative control, 0.2 and 0.4 mg/mL), berberine (5 and 10 μM), paeoniflorin (18.75 and 37.5 μM) for 24 h and then incubated with 700 μg/mL LPS for 24 h. After that, CCK-8 was used to measure cell viability. The morphology of the cells in each group was observed under an inverted microscope (Olympus, Tokyo, Japan).

### 4.10. Statistical Analysis

All data represent at least three independent biological replicates, expressed as mean ± standard deviation (SD). The sample size (n) per group was indicated in the figure legends. Statistical analysis was performed using GraphPad Prism 9.5.0. Group comparisons were made using One-Way ANOVA or Welch’s *t*-test, as appropriate. Multiple-comparison control was performed using Dunnett’s multiple-comparison test. Statistical significance was defined as *p* < 0.05 for all analyses.

## 5. Conclusions

**Figure 10:**
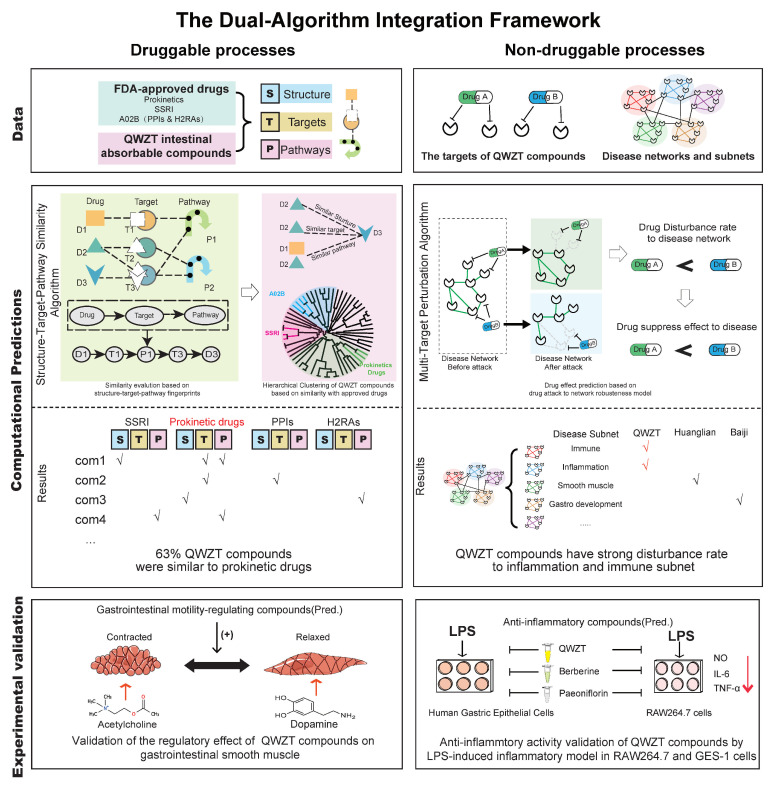
Schematic workflow of the dual-algorithm integration framework.

Our research aims to establish a solid foundation of scientific data to support the clinical application of QWZT. At the same time, we integrated the structure–target–pathway similarity algorithm and multi-target perturbation algorithm to identify the similar and specific compounds of TCM and FDA-approved drugs in the treatment of disease (Figure 10). QWZT can treat CG by regulating gastrointestinal smooth muscle and exerting anti-inflammatory effects. Finally, we experimentally validated the accuracy of our predictions. The proposed strategy aids in elucidating the complex mechanisms of TCM, providing valuable insights. This approach can be extended to other multi-compound drugs in the future, promoting their clinical application and advancing precision medicine.

## Figures and Tables

**Table 1:** List of QWZT intestinal absorbable compounds.

Compound	Source	Formula	RT (min)	Precursor Ion				Peak Area	Maximum Peak Area Ratio
				Type	Detected *m*/*z*	Expected *m*/*z*	Error (ppm)		
Magnoflorine	HL	C_20_H_23_NO_4_	20.20	[M + H]^+^	342.1696	342.1701	−1.4	1.67 × 10^11^	23.06%
Jatrorrhizine	HL	C_20_H_19_NO_4_	23.38	[M + H]^+^	338.1382	338.1386	−1.2	1.23 × 10^11^	16.93%
Berberine	HL	C_20_H_17_NO_4_	25.04	[M + H]^+^	336.1229	336.1232	−1.1	8.19 × 10^10^	11.29%
Epiberberine	HL	C_20_H_17_NO_4_	23.13	[M + H]^+^	336.1226	336.1232	−1.9	6.82 × 10^10^	9.40%
Sucrose	BS	C_12_H_22_O_11_	2.08	[M+Cl]^−^	377.0852	377.0846	1.5	4.51 × 10^9^	2.72%
Quinic acid	HL	C_7_H_12_O_6_	1.62	[M − H]^−^	191.0559	191.0553	2.8	4.42 × 10^9^	2.66%
Albiflorin	BS	C_23_H_28_O_11_	20.51	[M + H]^+^	481.1701	481.1706	−1.0	1.92 × 10^10^	2.64%
Palmatine	HL	C_21_H_21_NO_4_	24.08	[M + H]^+^	352.1539	352.1546	−2.0	1.75 × 10^10^	2.42%
Danshensu	HL	C_9_H_10_O_5_	16.93	[M − H − H_2_O]^−^	179.0348	179.0342	3.4	3.77 × 10^9^	2.27%
Cryptochlorogenic acid	HL	C_16_H_18_O_9_	18.22	[M − H]^−^	353.0873	353.0869	1.3	2.87 × 10^9^	1.73%
Oxypaeoniflorin	BS	C_23_H_28_O_12_	19.16	[M − H]^−^	495.1503	495.1498	1.1	2.34 × 10^9^	1.41%
p-Hydroxybenzaldehyde	BJ	C_7_H_6_O_2_	23.21	[M − H]^−^	121.0291	121.0285	4.3	1.96 × 10^9^	1.18%
Salicylic acid	BS, BJ	C_7_H_6_O_3_	23.80	[M − H]^−^	137.0241	137.0236	3.2	1.70 × 10^9^	1.03%
Oleanonic acid	BS, DY	C_30_H_46_O_3_	28.36	[M + H]^+^	455.3518	455.3523	−1.2	6.07 × 10^9^	0.84%
Pyrogallol	BS	C_6_H_6_O_3_	17.43	[M + H]^+^	127.0390	127.0394	−3.7	3.37 × 10^9^	0.46%
Protocatechuic acid	BJ	C_7_H_6_O_4_	17.02	[M − H]^−^	153.0191	153.0185	3.5	7.51 × 10^8^	0.45%
Methyl gallate	BS	C_8_H_8_O_5_	19.28	[M − H]^−^	183.0296	183.0291	2.8	7.36 × 10^8^	0.44%
Paeoniflorin	BS	C_23_H_28_O_11_	20.92	[M + H]^+^	498.1969	498.1973	−0.8	3.04 × 10^9^	0.42%
Benzoylpaeoniflorin	BS	C_30_H_32_O_12_	26.75	[M + H]^+^	602.2231	602.2232	−0.3	2.81 × 10^9^	0.39%
Epicatechin	DY	C_15_H_14_O_6_	19.36	[M − H]^−^	289.0714	289.0709	1.8	5.46 × 10^8^	0.33%
Ellagic acid	DY	C_14_H_6_O_8_	21.94	[M − H]^−^	300.9985	300.9980	1.7	4.15 × 10^8^	0.25%
Caffeic acid	HL	C_9_H_8_O_4_	17.16	[M − H]^−^	179.0347	179.0342	2.5	4.04 × 10^8^	0.24%
Manninotriose	JNJ	C_18_H_32_O_16_	2.58	[M − H]^−^	503.1612	503.1608	0.8	2.85 × 10^8^	0.17%
Ferulic acid	HL	C_10_H_10_O_4_	20.74	[M + H]^+^	195.0651	195.0657	−2.7	8.62 × 10^8^	0.12%
p-Coumaric acid	HL	C_9_H_8_O_3_	18.47	[M − H]^−^	163.0399	163.0394	3.3	1.49 × 10^8^	0.09%
Benzoic acid	BS	C_7_H_6_O_2_	20.34	[M + H]^+^	123.0438	123.0444	−4.2	3.81 × 10^8^	0.05%
Isovanillin	HL	C_8_H_8_O_3_	21.47	[M + H]^+^	153.0546	153.0551	−3.1	3.78 × 10^8^	0.05%
Cianidanol	BS	C_15_H_14_O_6_	20.25	[M − H]^−^	289.0714	289.0709	1.7	4.2 × 10^7^	0.03%
Kaempferol	BS	C_15_H_10_O_6_	28.04	[M − H]^−^	285.0402	285.0396	1.9	2.60 × 10^7^	0.02%
Rosamultin	DY	C_36_H_58_O_10_	27.65	[M − H]^−^	685.3722	685.3717	0.8	2.43 × 10^7^	0.01%
Coumarin	HL	C_9_H_6_O_2_	20.67	[M + H]^+^	147.0440	147.0445	−3.8	8.76 × 10^7^	0.01%
Flavanone	DY	C_15_H_12_O_2_	24.01	[M + H]^+^	225.0907	225.0912	−2.4	6.17 × 10^7^	0.01%
Naringenin	HL	C_15_H_12_O_5_	23.95	[M + H]^+^	273.0754	273.0760	−2.2	5.44 × 10^7^	0.01%

Abbreviations: HL, Huanglian; BS, Baishao; BJ, Baiji; DY, Diyu; JNJ, Jineijin. The detected and expected *m*/*z* value corresponds to the relevant precursor ion.

**Table 2:** Potential regulatory effects on gastrointestinal smooth muscle of QWZT compounds.

Compound	Maximum Peak Area Ratio	Regulation of Gastrointestinal Smooth Muscle	Treatment of Chronic Gastritis
Magnoflorine	23.06%	—	—
Jatrorrhizine	16.93%	Increase the contractions of ileum longitudinal muscles [14]	Suppress the inflammation and colonization of *H. pylori* in CAG rats [15]
Berberine	11.29%	Inhibit the contractility of isolated gastric intestine smooth muscle [16]	Improve CAG via alleviating inflammation response [17]
Epiberberine	9.40%	—	Reduce gastric inflammation caused by *H. pylori* infection [18]
Palmatine	2.42%	—	Alleviate the histological damage of gastric mucosa and inflammatory response [19]

## Data Availability

Data presented in this study is contained within the article and Supplementary Material. Further inquiries can be directed to the corresponding author.

## Supplementary Materials

The following supporting information can be down-loaded at: https://www.mdpi.com/article/10.3390/ph18111743/s1, Figure S1: PPI network of six gastritis-related diseases; Figure S2: Quality control of the clustering results of FDA-approved drugs; Figure S3: GO biological process analysis of FDA-approved drugs; Figure S4: The inflammation functional subnetwork of diseases involving QWZT intestinal absorbable compounds; Table S1: Similarity distance matrix of QWZT compounds and FDA-approved drugs; Table S2: Summary of similar compounds between QWZT and FDA-approved drugs; Table S3: The Z-score of potentially similar compounds; Table S4: QWZT compounds involved in inflammation functional subnetworks; Table S5: Potentially specific compounds (The literature reporting the anti-inflammatory activity of the drug is as follows [55,56,57,58,59,60,61,62,63,64,65,66,67,68,69,70,71,72,73]. The literature reporting on drug treatments for chronic gastritis is as follows [15,74,75,76,77,78,79]).

## Abbreviations

The following abbreviations are used in this manuscript:

AD	average degree
ASPL	average shortest path length
BP	biological process
CG	chronic gastritis
CC	closeness centrality
DC	degree centrality
DRD2	dopamine receptor D2
GES-1	Human Gastric Epithelial Cells
GO	Gene Ontology
H2RAs	H2 receptor antagonists
LPS	lipopolysaccharide
MOA	mechanisms of action
PPI	protein–protein interaction
PPIs	proton pump inhibitors
QWZT	Qing-Wei-Zhi-Tong micro-pills
SSRIs	selective serotonin reuptake inhibitors
TCM	traditional Chinese medicine
TCM-NP	traditional Chinese medicine network pharmacology
TNF	tumor necrosis factor
UPLC-MS	Ultra Performance Liquid Chromatography–Mass Spectrometry
