# Supplementary material for: Dual-Algorithm Integration Framework Reveals Qing-Wei-Zhi-Tong’s Dual Mechanisms in Chronic Gastritis
Source: Pharmaceuticals (Basel). 2025 Nov 17;18(11):1743. doi: 10.3390/ph18111743 (PMC12655216; doi:10.3390/ph18111743)
Supplement: Supplementary file 1 [file pharmaceuticals-18-01743-s001.zip › Supplementary Figures.pdf]

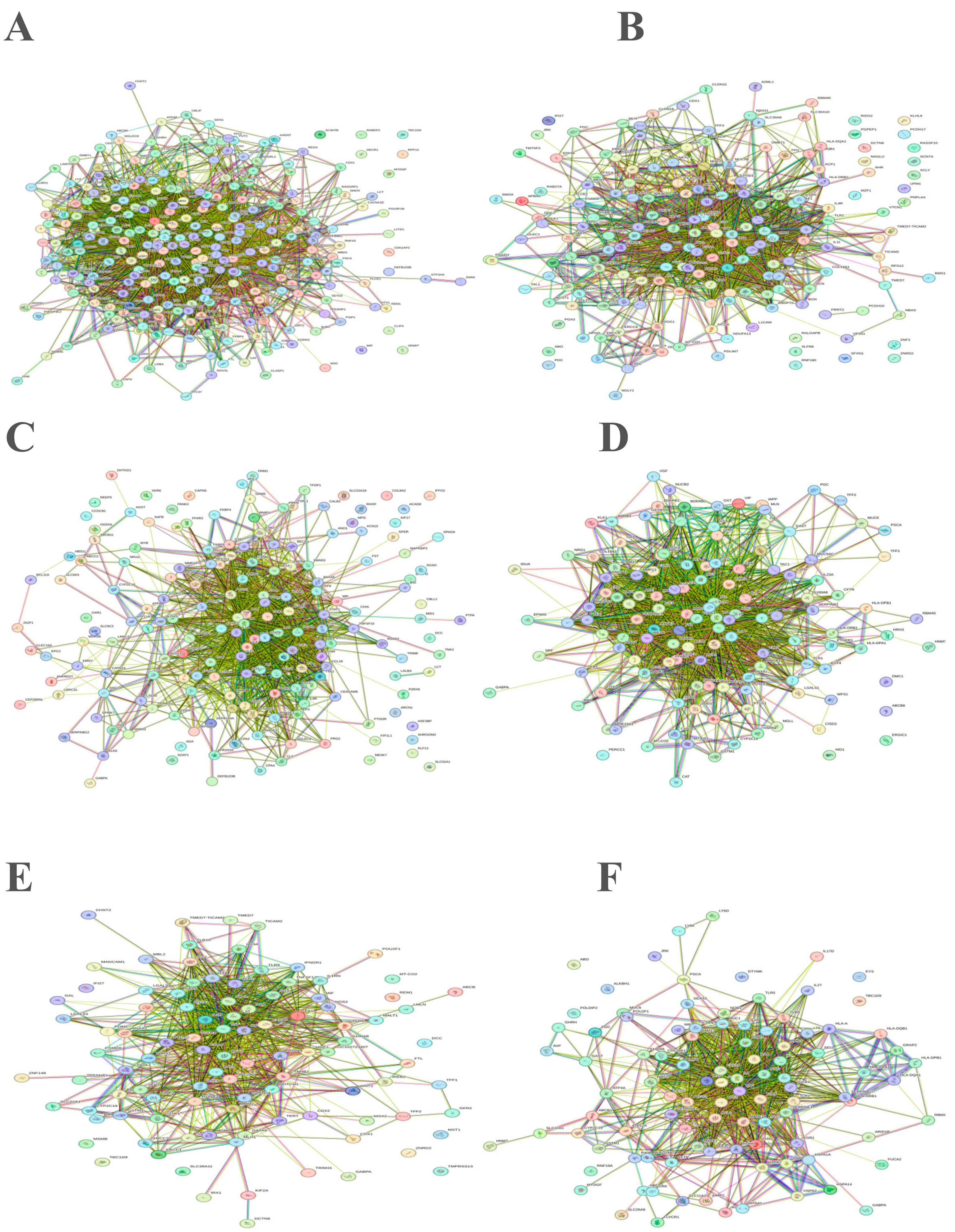

Figure S1. The PPI network of six gastritis-related diseases.

(A) The PPI network of Gastritis (271 nodes, 4625 edges).

(B) The PPI network of Gastritis Atrophic (180 nodes, 1799 edges).

(C) The PPI network of Eosinophilic Esophagitis (175 nodes, 1507 edges).

(D) The PPI network of Gastric Ulcer (130 nodes, 1897 edges).

(E) The PPI network of Chronic Gastritis (101 node, 852 edges).

(F) The PPI network of Duodenal Ulcer (100 nodes, 905 edges).

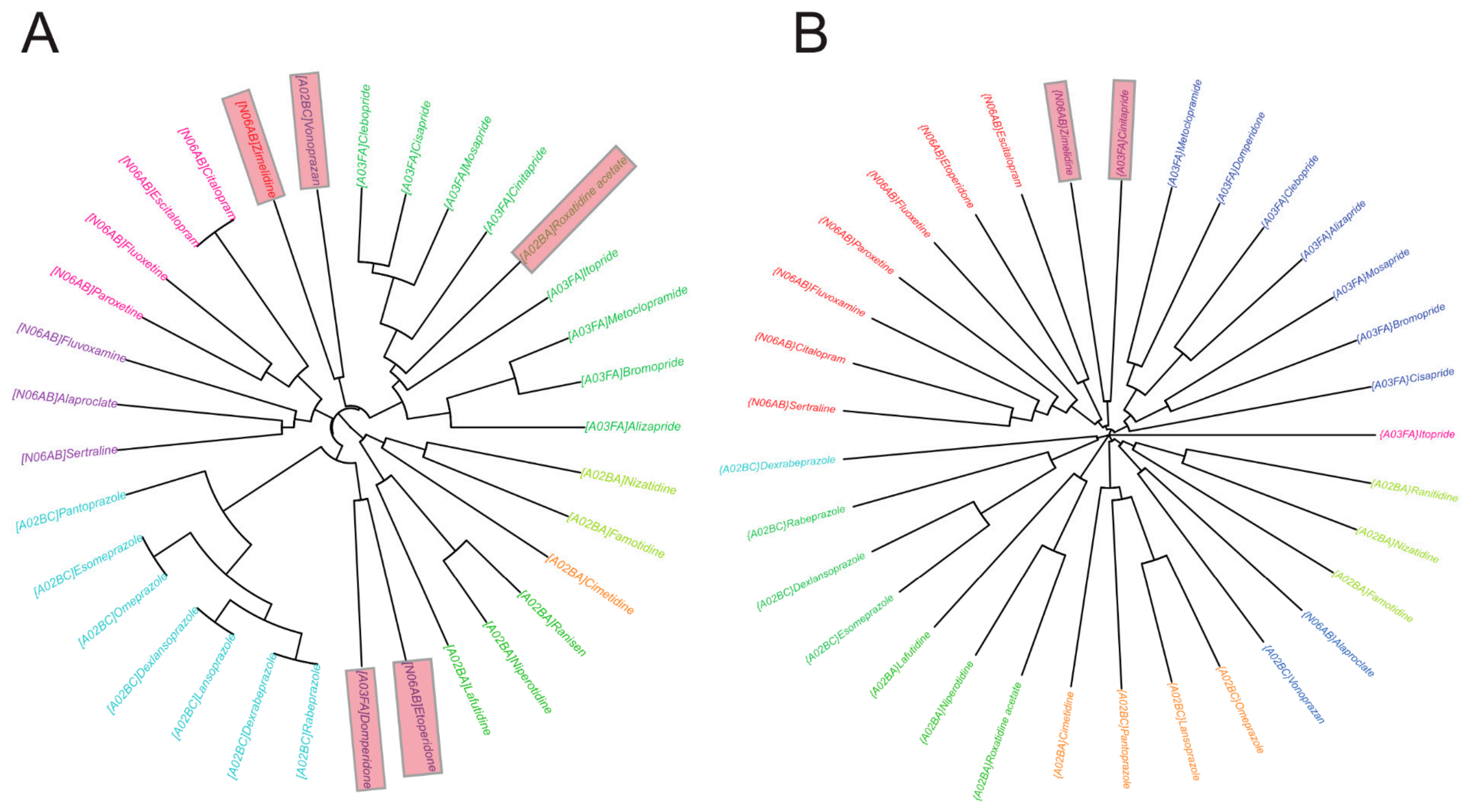

Figure S2. Quality control of the clustering results of FDA-approved drugs.

(A) Unsupervised hierarchical clustering of FDA-approved drugs based on similarity of chemical structure.

(B) Unsupervised hierarchical clustering of FDA-approved drugs based on similarity of targets.

FDA-approved drugs with red rectangles around them indicate that clustering is not accurate for their corresponding categories.

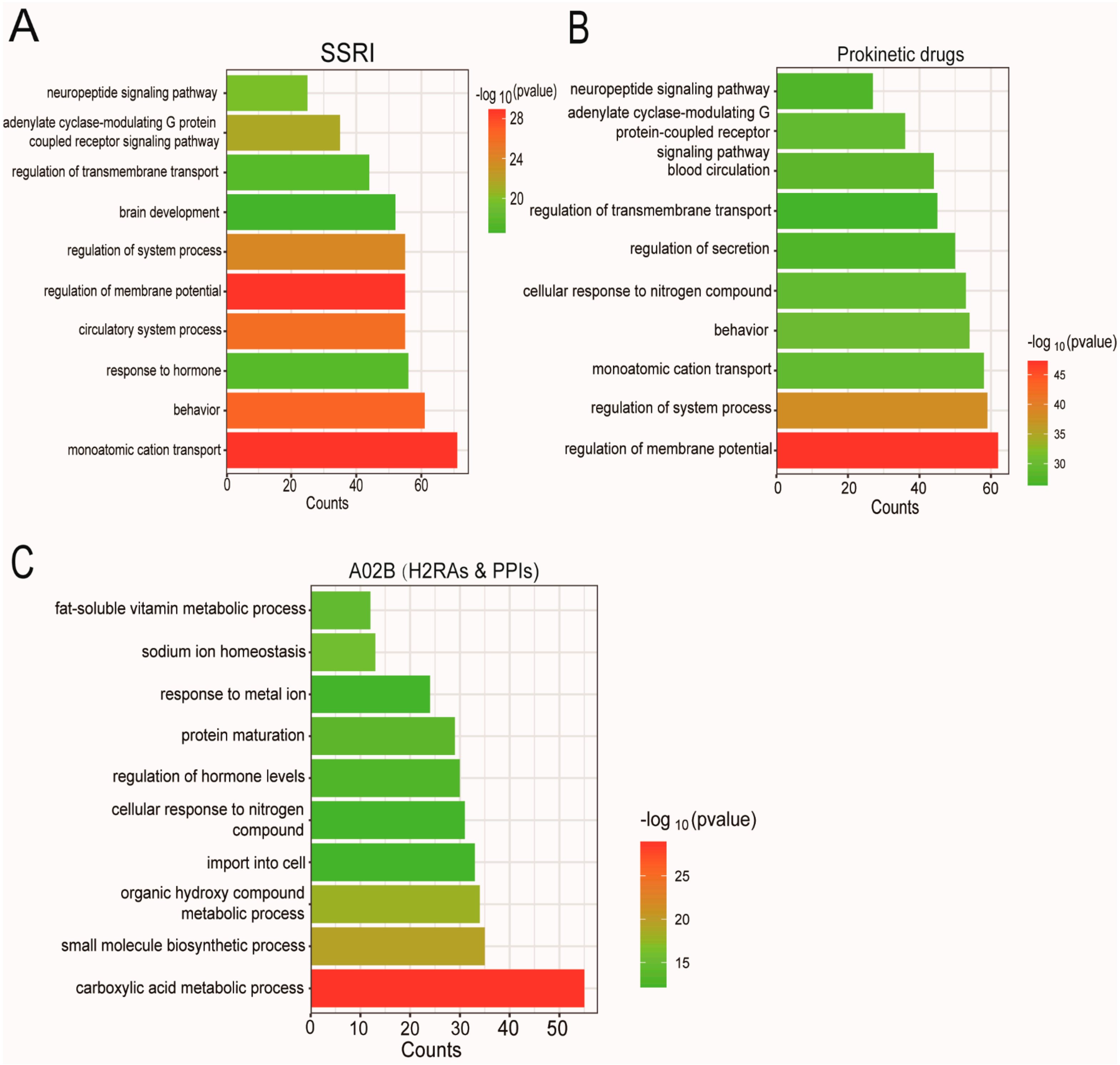

Figure S3. GO biological process analysis of FDA-approved drugs.

(A) Top 10 GO terms in SSRI.

(B) Top 10 GO terms in the common targets of H2RAs and PPIs.

(C) Top 10 GO terms in Prokinetic drugs.

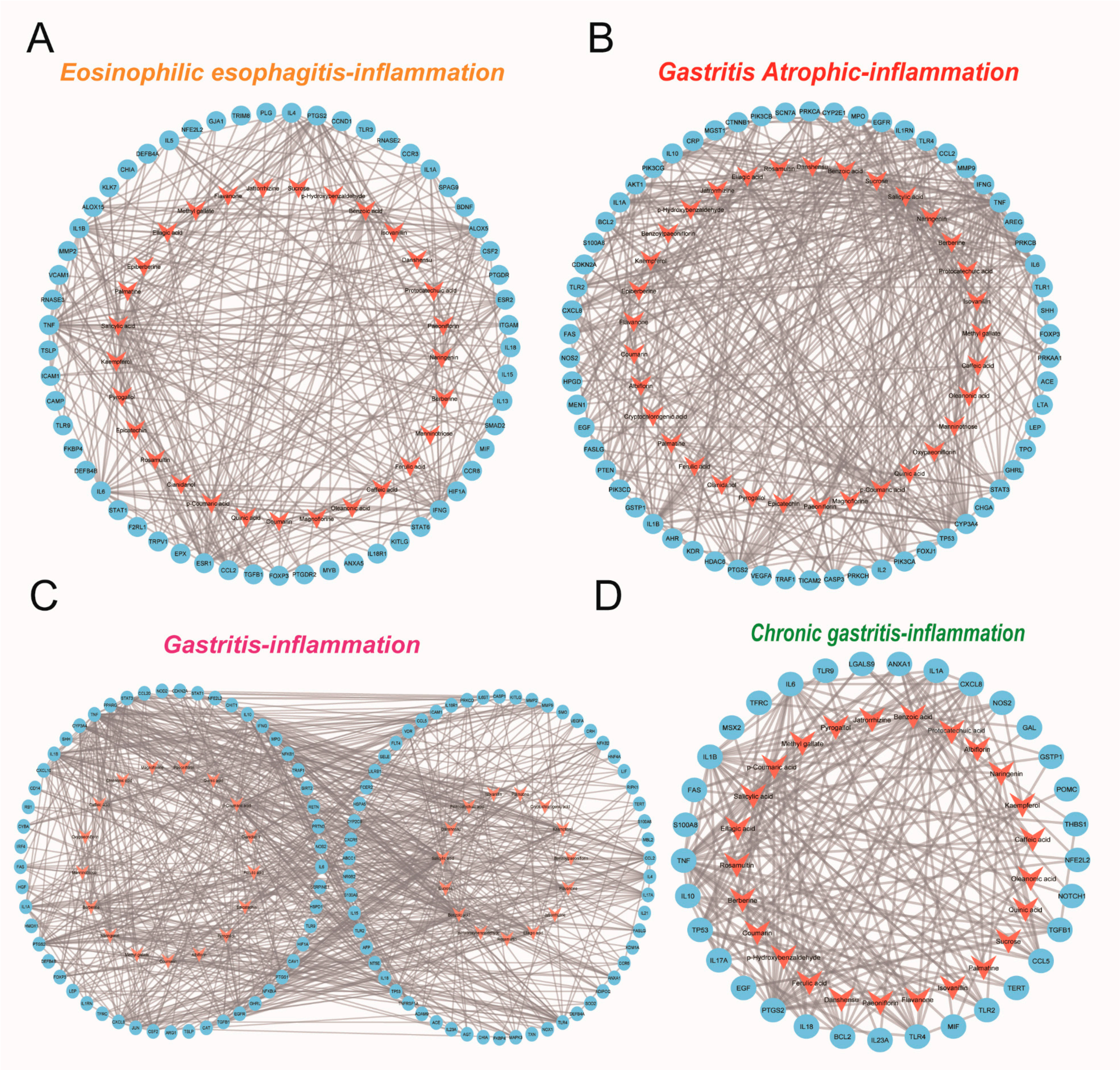

Figure S4. The inflammation functional subnetwork of diseases involving QWZT intestinal absorbable compounds

(A) QWZT intestinal absorbable compounds- eosinophilic esophagitis-inflammation network.

(B) QWZT intestinal absorbable compounds-gastritis atrophic- inflammation network.

(C) QWZT intestinal absorbable compounds-gastritis-inflammation network.

(D) QWZT intestinal absorbable compounds-chronic gastritis-inflammation network.
